# Genome-Enabled Prediction of Breeding Values for Feedlot Average Daily Weight Gain in Nelore Cattle

**DOI:** 10.1534/g3.117.041442

**Published:** 2017-04-07

**Authors:** Adriana L. Somavilla, Luciana C. A. Regitano, Guilherme J. M. Rosa, Fabiana B. Mokry, Mauricio A. Mudadu, Polyana C. Tizioto, Priscila S. N. Oliveira, Marcela M. Souza, Luiz L. Coutinho, Danísio P. Munari

**Affiliations:** * Faculdade de Ciências Agrárias e Veterinárias, Universidade Estadual Paulista, Jaboticabal, Brazil; † Embrapa Pecuária Sudeste, São Carlos, Brazil; ‡ University of Wisconsin–Madison, Wisconsin; § Departmento de Genética e Evolução, Universidade Federal de São Carlos, Brazil; ** Departmento de Zootecnia, Universidade de São Paulo, Piracicaba, Brazil

**Keywords:** genomic selection, *Bos taurus indicus*, growth, feedlot performance, GenPred, Shared data resources

## Abstract

Nelore is the most economically important cattle breed in Brazil, and the use of genetically improved animals has contributed to increased beef production efficiency. The Brazilian beef feedlot industry has grown considerably in the last decade, so the selection of animals with higher growth rates on feedlot has become quite important. Genomic selection (GS) could be used to reduce generation intervals and improve the rate of genetic gains. The aim of this study was to evaluate the prediction of genomic-estimated breeding values (GEBV) for average daily weight gain (ADG) in 718 feedlot-finished Nelore steers. Analyses of three Bayesian model specifications [Bayesian GBLUP (BGBLUP), BayesA, and BayesCπ] were performed with four genotype panels [Illumina BovineHD BeadChip, TagSNPs, and GeneSeek High- and Low-density indicus (HDi and LDi, respectively)]. Estimates of Pearson correlations, regression coefficients, and mean squared errors were used to assess accuracy and bias of predictions. Overall, the BayesCπ model resulted in less biased predictions. Accuracies ranged from 0.18 to 0.27, which are reasonable values given the heritability estimates (from 0.40 to 0.44) and sample size (568 animals in the training population). Furthermore, results from *Bos taurus indicus* panels were as informative as those from Illumina BovineHD, indicating that they could be used to implement GS at lower costs.

Brazil has the world’s second largest cattle herd with over 200 million heads (Instituto Brasileiro de Geografia e Estatística 2013), with the Nelore (*Bos taurus indicus*) being the most widespread and economically important breed. As the total pasture area in Brazil has decreased over the decades, productivity gains have become an important factor for beef production (Martha *et al.* 2012). The Nelore breed has been selected for growth rate traits on pasture based on traditional pedigree and phenotype analysis; however, the Brazilian beef feedlot industry has grown about 50% in the last decade (Millen *et al.* 2011), and novel breeding objectives and criteria are required.

In this context, the application of technologies to improve animal performance and thus to supply genetically improved animals for both pasture and feedlot systems are a critical factor to overcome the challenge of increasing Brazilian beef production efficiency. Nowadays, exploring the availability of technology to genotype thousands of single nucleotide polymorphisms (SNPs) distributed across the genome allows the application of GS. Phenotypic and SNP data information are then combined to predict GEBV earlier in the life of the animals (Meuwissen *et al.* 2001). It has been argued that GS could lead to a decrease in generation interval, an improvement of the rate of genetic gain (Schaeffer 2006), and also assist the better control of inbreeding rates (Daetwyler *et al.* 2007).

Based on the importance of the Nelore cattle in Brazil and the increasing use of feedlot systems, it is necessary to identify appropriate methodologies that allow GS of animals with higher growth rates on feedlots. The aim of the current study was to compare different regression models and SNP panels in terms of accuracy, bias, and precision of GEBV for ADG in feedlot-finished Nelore steers.

## Materials and Methods

### Samples

During the mating seasons of 2006/07 through 2008/09, 804 steers, offspring of 34 Nelore bulls from 17 lineages, chosen to represent the genealogies of the Nelore breed in Brazil, were generated through fixed-time artificial insemination in five farms. They were raised to 21 months of age and then moved to either the Embrapa Southeast Livestock (São Carlos, SP, Brazil) or the Embrapa National Beef Cattle Center (Campo Grande, MS, Brazil) during three seasons in feedlot experiment periods (2009, 2010, and 2011). Animals were fed with a total mixed ration diet with 13% crude protein and 71% total digestible nutrients (dry matter basis, corn or sorghum, soybean meal, soybean hull, cotton seed, limestone, mineral mixture, urea, and monensin). The diet was provided twice a day in which the feed offered (total mixture composed by concentrate:silage, 40:60 ratio) was adjusted daily *ad libitum*. The animals were weighed every 14 d without fasting, for an average period of 91 d. Steer rearing and sample collection protocols were approved by the Animal Care and Use Committee from the Embrapa Southeast Livestock.

### Phenotype and genotype datasets

The initial dataset consisted of 7236 weight records from the 804 steers, but only those from the 15th up to 77th d in feedlot were considered to estimate ADG, to disregard the first weight, and also because after this period >30% of the animals had already been slaughtered. A linear regression analysis of live weight over time was performed using the remained 3523 records from 803 steers, using the lm function of the R software (R Development Core Team 2014). The slope was used as the ADG during the feedlot period for the purpose of considering only the linear weight gain and avoiding comparison with different feedlot period lengths.

Steers were assigned to 39 contemporary groups (CG) containing from 5 to 42 animals, which combined information on mating season (three levels), experimental feedlot (two levels), and slaughter group (32 levels of animals slaughtered in the same week). After that, the phenotype and genotype datasets were merged to ensure that they had the same individuals. The summary of age at feedlot entry, starting weight, ADG, and days in feedlot on the remaining animals are presented in Table 1.

**Table 1:** Summary of age and weight at feedlot entry, ADG, and days in feedlot for the 718 Nelore steers

	Age (d)	Weight (kg)	ADG (kg/d)	Days in Feedlot
Minimum	542	226	0.193	48
Mean (± SD)	649 (45)	361 (51)	1.235 (0.407)	92 (20)
Maximum	745	510	2.457	119

ADG, average daily weight gain.

In total, 780 steers and 34 bulls were genotyped with the Illumina BovineHD BeadChip (Illumina, San Diego, CA). The initial dataset contained 742,906 markers, in which unplaced, mitochondrial, and sex-linked SNPs were first discarded, as well as duplicated markers (*e.g.*, two different names and positions for the same SNP). SNPs were also filtered based on two other panels: GeneSeek Genomic Profiler (GGP) HDi 80K and GGP LDi 20K (Gene Seek Inc., Lincoln, NE). The panels were built specifically for *B. taurus indicus* breeds. Originally, the GGP HDi 80k/LDi 20k contained 74,085/19,721 markers, of which 69,942/18,464 were available in the primary dataset.

Paternity correction and quality control (QC) were performed to improve results. To deal with SNPs presenting significant deviation from the Hardy–Weinberg Proportions (HWP) deviation, we checked plots of HWP *vs.* percentage of heterozygous, and 17 SNPs with >80% of heterozygous were excluded from the three datasets because they could reflect an error during the genotyping procedure (Ziegler 2009). QC was performed using the R package SNPtats (Clayton 2015). SNPs were kept for further analysis only if they had call rate >98% and minor allele frequency (MAF) >1%. The MAF filter excluded 20.0, 1.9, and 7.3% of the total SNPs from the 770k, HDi, and LDi panels, respectively.

After QC, the Beagle v.3.3.2 (Browning and Browning 2009) software was used for phase inference and imputation of missing genotypes for each SNP panel. Finally, to constitute a fourth SNP panel scenario, Tagger (Bakker *et al.* 2005), which is based on linkage disequilibrium (LD) between markers (*r^2^*), was used. This tool estimates the *r^2^* between all SNP pairs and then selects a minimal set (TagSNPs) of markers with a *r^2^* ≥ 0.3 with at least one another marker on the same chromosome. We have chosen this threshold because it is the overall average *r^2^* at the distance of 10–25 kb, obtained in a previous analysis of the same animals (Mudadu *et al.* 2016). The final number of SNP was 15,863, 63,945, 82,933, and 534,787 for the LDi, HDi, TagSNP, and 770k panels, respectively.

### Fixed effects modeling and adjusted phenotypes

The adjusted phenotype ($\hat{y}$) was represented as $\hat{y}=y-\;1\hat{\mu }-W\hat{\alpha },$ in which y is the vector of observations, $\hat{\mu }$ is the overall mean, W is an incidence matrix for fixed effects (CG and animal age at feedlot entry), and $\hat{\alpha }$ is the vector of fixed effects estimates. A residual analysis was performed at this point and animals with the normalized residuals with absolute values >3.5 were removed, thus 718 steers remained in the dataset.

### Models for genomic-enabled prediction

Three specifications were considered for building genome-enabled prediction models: BayesA, BayesCπ, and BGBLUP. The R package BGLR (de los Campos and Rodriguez 2016) was used to fit the models, a flat (noninformative) prior was assigned to the intercept. For the BayesA method, a normal distribution was assigned to the marker effects, $\beta_{j}\;∼\;N\left(0,\sigma_{\beta j}^{2}\right),$ where j=(1,…,p), p is the number of SNPs, and $\sigma_{\beta j}^{2}$ is the individual variance for the SNP effect. In a second level of hierarchy, each $\sigma_{\beta j}^{2}$ was assigned independent and identically distributed (iid) Scaled-inverse χ^2^ density, with degrees of freedom ($df_{\beta }$) set to 5 and scale parameter ($S_{\beta }$) treated as unknown, following a γ distribution with shape (s) and rate (r) parameters. The parameter *s* was set to *s* = 1.1 and *r* was solved so that 80% of proportion of the variance of the response was attributed the linear predictor. On this model, the prior marginal distribution of marker effects is a scaled-t density, with parameters $df_{\beta }$ and $S_{\beta }$ (Rosa *et al.* 2003).

For the BayesCπ model, the prior for each marker effect was an iid mixture of point of mass (1 −  π) at zero (spike) and a slab that follows a Gaussian distribution, $\;\beta_{j}\;∼\;N\left(0,\sigma_{\beta }^{2}\right)\pi ,$ where $\sigma_{\beta }^{2}$ is the common variance for the SNP effects. The additional parameter π represents the prior proportion of nonzero effects and was treated as an unknown, with a β prior distribution $\pi \;∼\beta \left(p_{0},\pi_{0}\right),$ with $\;p_{0}>0\;\mathrm{and}\;\pi_{0}\in \left[0,1\right].$ The parameters were set to $p_{0}=2$ and $\pi_{0}=0.5,$ which gave a uniform prior in the interval [0,1]. Thus, differently from BayesA, BayesCπ sets some SNP effects to zero, within a variable selection framework.

The BGBLUP model was implemented as a Bayesian Reproducing Kernel Hilbert Spaces regression (de los Campos *et al.* 2009), using a single kernel, user-defined (co)variance matrix K. The vectors of additive random effects were assigned multivariate normal priors, $u\;∼\;N\left(0,K\sigma_{u}^{2}\right),$ in which $\sigma_{u}^{2}\;∼\;\chi^{-2}\left(S,\mathrm{df}\right)$ and K was set as a marker-derived relationship matrix G, built as the first method proposed by VanRaden (2008). Briefly, let $M_{nxm}$ be a genotype matrix with n (number of samples) rows and m (number of SNPs) columns, $Z_{nxm}$ be the centered M matrix, and $G=\;ZZ^{\prime }/\left[2\sum p_{j}\left(1-p_{j}\right)\right],$ where the denominator is the total variance across loci. The degrees of freedom (df) was set to 5 and the scale parameter (S) was solved so that 80% of proportion of the variance of the response was attributed the linear predictor.

The number of iterations, burn-in, and thinning interval parameters were graphically evaluated and were different for each model (Table 2), and the length of the chain used to compute posterior statistics was 25,000, 20,000, and 10,000 for BayesA, BayesCπ, and BGBLUP, respectively. For BayesA and BayesCπ, the marker-based genetic variance $\left(\sigma_{g}^{2}\right)$ was computed as the sum of the variance explained by each SNP marker $\left(\sigma_{\beta j}^{2}\right),$ while for BGBLUP the genetic variance was equal to $\sigma_{u}^{2}.\;$ For the three models, the narrow sense heritability was estimated as: $h^{2}=\sigma_{g}^{2}/\left(\sigma_{g}^{2}+\sigma_{e}^{2}\right),$ where $\sigma_{e}^{2}$ is the residual variance.

**Table 2:** Parameters of Gibbs sampler for each model

MCMC Samples	Model		
	BayesA	BayesCπ	BGBLUP
Total	400,000	600,000	160,000
Burn-in	150,000	200,000	60,000
Thinning	10	20	10
Posterior*^a^*	25,000	20,000	10,000

MCMC, Markov chain Monte Carlo; BGBLUP, Bayesian genomic best linear unbiased prediction.

a Final number of samples used to calculate features of posterior distributions.

### Validation

The dataset was divided into training (animals from seasons 1 and 2) and testing (animals from season 3) subgroups, which contained 568 and 150 animals, respectively. For the BayesA and BayesCπ models, the GEBV on the testing set was defined as $\mathrm{GEB}V_{i\left(\mathrm{tst}\right)}=\;\sum_{j=1}^{p}g_{ij}{\hat{\beta }}_{\mathrm{trn}},$ where $g_{ij}$ is the genotype of the *j*th SNP on the *i*th animal and ${\hat{\beta }}_{\mathrm{trn}}$ is the vector of the SNP marker effect estimated on the training set. For BGBLUP, phenotypes of the testing subgroup were set as missing and samples of u were obtained in each iteration from the posterior distribution $\left[u,\sigma_{u}^{2},\sigma_{e}^{2}|\hat{y}\right].$

The correlation between GEBV and the adjusted phenotype of animals on testing subgroup, r(GEBVi(tst),yi(tst)^), was used as an estimation of prediction accuracy. The slopes of regressing adjusted phenotypes on GEBV for animals in the testing subgroup (bytst,^GEBVtst,) were evaluated as a measure of bias, which can be used to verify whether genomic predictions are inflated or deflated. The last comparison criterion was the mean square error, $\mathrm{MSE}=\;\sum_{1}^{\mathrm{ntst}}{\left(\mathrm{GEB}V_{i}-\;{\hat{y}}_{i}\right)}^{2}/\left(n_{\mathrm{tst}}\right),$ where $n_{\mathrm{tst}}$ is the size of testing dataset, that was used as a measure of precision and bias of the point estimator.

### Data availability

The phenotypic and genotypic data are available at the figshare repository and their description and accession numbers are listed in Supplemental Material, File S1. File S2 contains a custom R script used in the analysis. 

## Results and Discussion

### Accuracy of GEBV

Pearson correlation coefficients between adjusted phenotypes and GEBV were used as a proxy of genome-enabled prediction accuracies (Table 3). All estimates were quite similar, ranging from 0.24 to 0.27. Bolormaa *et al.* (2013) reported even lower accuracies (from 0.13 to 0.24) of GEBV for ADG in feedlot using GBLUP estimates in *B. taurus taurus* and *B. taurus indicus* animals. When analyzing ADG of almost 4000 Nelore young bulls in pasture using traditional BLUP, Fragomeni *et al.* (2013) reported an EBV accuracy of 0.56, which suggests that we could achieve higher accuracies than we found in the present study.

**Table 3:** Pearson correlation coefficients used as proxy estimates of prediction accuracies of GEBV for ADG of the 150 animals in the testing subgroup

Model	SNP Panel*^a^*			
	770k	TagSNP	HDi	LDi
BGBLUP	0.26	0.24	0.25	0.26
BayesA	0.26	0.25	0.26	0.27
BayesCπ	0.26	0.25	0.25	0.26

SNP, single nucleotide polymorphism; HDi, high-density indicus; LDi, low-density indicus; BGBLUP, Bayesian genomic best linear unbiased prediction.

a Actual number of SNPs included in the analysis: 770k, 534,787; TagSNP, 82,933; HDi, 63,945; and LDi, 15,863.

It is known that the success of GS depends on the accuracy of GEBV, which in turn is a function of heritability, size of training population, and effective population size (*N_e_*) (Goddard and Hayes 2009). Based on the simulation presented by van der Werf (2013), who considered a population with *N_e_* = 250 (estimated *N_e_* of Nelore cattle = 214 (Mudadu *et al.* 2016)) and a trait with *h*^2^ = 0.5, a training population of 500 animals would reach an accuracy of 0.2, similar to our results. Moreover, the authors showed that a training population of >2000 individuals would be required to achieve an accuracy of 0.4. Another key factor is the level of relationship among animals in the training and testing sets. The present study evaluated half-sib families and, according to Hayes *et al.* (2009), this structure only allows estimation of the effects of paternal alleles with high accuracy, decreasing the reliability of the GEBVs.

Taking into account the above-mentioned factors, we point out that the crucial points would be to increase the number of reference animals and to include animals with different levels of relationship, thus the SNP marker effects could be better estimated. Since ADG in feedlot-finished steers could be viewed as a new selection criterion for Nelore cattle, it is important to estimate the GEBVs with high accuracy in order to allow the selection of young animals and genetic gains at reduced genotyping costs.

### Bias and precision measures of GEBVs

Regression coefficients of adjusted phenotypes on GEBV (Table 4) were used to measure the extent of prediction bias, since values greater or lower than one are related to deflated or inflated GEBV, respectively. For the 770k panel, only the results from BayesCπ models were not considered to be biased. Also, it is clear that estimates from BayesA models (except for TagSNP) were deflated, which means that the GEBVs were not in the same scale as the adjusted phenotypes. The opposite was observed for all models applied to the TagSNP dataset, thus it seems that selecting markers based only on their pairwise *r*^2^ resulted in overestimated predictors.

**Table 4:** Regression coefficients (*b*) of GEBV on adjusted phenotype and MSE of predictions for the 150 animals in testing subgroup

Model	SNP Panel*^a^*							
	770k		TagSNP		HDi		LDi	
	*b*	MSE	*b*	MSE	*b*	MSE	*b*	MSE
BGBLUP	1.15	1.58	0.46	1.59	1.10	1.58	1.11	1.59
BayesA	1.29	1.09	0.69	1.24	1.68	1.32	1.99	1.37
BayesCπ	0.98	1.12	0.45	1.12	0.94	0.94	0.93	0.94

SNP, single nucleotide polymorphism; HDi, high-density indicus; LDi, low-density indicus; *b*, regression coefficient; MSE, mean squared errors; BGBLUP, Bayesian genomic best linear unbiased prediction.

a Actual number of SNPs included in the analysis: 770k, 534,787; TagSNP, 82,933; HDi, 63,945; and LDi, 15,863.

Differences among prediction accuracies were negligible, thus information on slopes and mean squared errors (MSE) (Table 4) were combined and the models resulting in less biased GEBV were 770k-BayesCπ, HDi-BayesCπ, and LDi-BayesCπ. The current average cost of genotyping can easily reach $150.00, $100.00, and $50.00 per animal for 770k, HDi, and LDi, respectively. Therefore, if it was possible to predict accurate GEBV using less dense panels of SNPs at lower cost, the implementation and application of GS would be better accepted by the beef cattle industry.

### Estimates of variance components

The divergences in the variance components (Table 5) were expected, since the markers included in each model capture different proportions of the genetic variance. For example, the marker-based genetic variance estimated using BGBLUP was the lowest (about 0.02) in this study. For BayesA and BayesCπ, the genetic variance is a function of SNP effects and their uncertainty variances and allelic frequencies (Gianola *et al.* 2009). Results from BayesA models were not consistent among SNP panels and we hypothesized that, by fitting a larger number of markers, the captured marker-based genetic variance is greater (Table 5).

**Table 5:** Estimates of residual ($\sigma_{e}^{2}$) and genetic ($\sigma_{g}^{2}$) variance components, heritability ($h^{2}$), and proportion of nonzero effects (π) for all models

SNP Panel*^a^*	Parameter	BGBLUP*^b^*	BayesA*^b^*^,^*^c^*	BayesCπ*^b^*^,^*^c^*
770k	$\sigma_{e}^{2}$	0.05 (0.04–0.06)	0.06 (0.05–0.07)	0.05 (0.04–0.06)
	$\sigma_{g}^{2}$	0.02 (0.01–0.04)	0.06	0.03
	$h^{2}$	0.31 (0.19–0.45)	0.53 (0.49–0.58)	0.41 (0.36–0.47)
	π	―	―	0.98 (0.96–1.00)
TagSNP	$\sigma_{e}^{2}$	0.05 (0.04–0.06)	0.06 (0.05–0.07)	0.05 (0.04–0.06)
	$\sigma_{g}^{2}$	0.02 (0.01–0.04)	0.04	0.03
	$h^{2}$	0.32 (0.19–0.46)	0.40 (0.36–0.45)	0.42 (0.37–0.48)
	π	―	―	0.98 (0.96–1.00)
HDi	$\sigma_{e}^{2}$	0.05 (0.04–0.06)	0.06 (0.05–0.07)	0.05 (0.04–0.06)
	$\sigma_{g}^{2}$	0.02 (0.01–0.04)	0.03	0.03
	$h^{2}$	0.32 (0.19–0.46)	0.31 (0.28–0.35)	0.42 (0.37–0.48)
	π	―	―	0.98 (0.96–1.00)
LDi	$\sigma_{e}^{2}$	0.05 (0.04–0.06)	0.06 (0.05–0.07)	0.05 (0.03–0.06)
	$\sigma_{g}^{2}$	0.02 (0.01–0.04)	0.02	0.04
	$h^{2}$	0.32 (0.19–0.45)	0.28 (0.25–0.32)	0.44 (0.36–0.47)
	π	―	―	0.98 (0.96–1.00)

SNP, single nucleotide polymorphism; HDi, high-density indicus; LDi, low-density indicus; BGBLUP, Bayesian genomic best linear unbiased prediction; HPD, highest posterior density intervals.

a Actual number of SNPs included in the analysis: 770k, 534,787; TagSNP, 82,933; HDi, 63,945; and LDi, 15,863.

b Numbers in brackets refers to the HPD at 95% (lower bound–upper bound).

c HPD for $\sigma_{g}^{2}$ for models BayesA and BayesCπ could not be estimated.

BayesCπ models resulted in less biased GEBVs, and its coefficients of heritability ranged from 0.41 to 0.44 (Table 5). This was similar to the coefficient reported by Olivieri *et al.* (2016) for ADG in Nelore cattle in a postweaning feedlot performance test $\left(h^{2}=0.43\right).$ Although heritability is a population parameter, it is known that magnitudes of heritability estimates of similar traits are often similar across populations.

### Conclusions

For the purpose of comparing GEBV estimates using different SNP panels and Bayesian models, we considered some of the most common criteria used to evaluate the quality of the genome-enabled predictions. Overall, all SNP panels and models provided similar accuracies; however, *B. taurus indicus* SNP chips (HDi and LDi) and methods that zero a proportion of the SNP effects, such as BayesCπ, seem to result in less biased predictions. Furthermore, results from less dense marker panels based on *B. taurus indicus* were as good as the high-density panel, and at lower genotyping costs.

## Supplementary Material

Supplemental material is available online at www.g3journal.org/lookup/suppl/doi:10.1534/g3.117.041442/-/DC1.
