# Supplementary material for: Genome-Enabled Prediction of Breeding Values for Feedlot Average Daily Weight Gain in Nelore Cattle
Source: G3 (Bethesda). 2017 Apr 7;7(6):1855–9. doi: 10.1534/g3.117.041442 (PMC5473763; doi:10.1534/g3.117.041442)
Supplement: Supplementary file 1 [file 1855FileS1.pdf]

Description and accession of phenotypic and genotypic data

AdjustedADGs.txt

The first column contain the Animal ID (from 1 to 718), the second column refers to the year of birth during the study (1, 2 or 3), and the third column contains the adjusted feedlot average daily weight gain (adjADG), described in the "Material and methods" section.

Genotype files

The genotypes after the quality control (described in the "Material and methods" section) were made available as they were used in the analysis (Plink format). Each dataset contains three files with the following extensions: *.bed, *.bim, *.fam. File S2 contains a custom script describing the analysis and how these files were used. Because this is a prediction study, there is no information on SNPs positions.

The files can be accessed in: https://figshare.com/articles/Genome-enabled_prediction_for_feedlot_average_daily_gain_in_Nelore_cattle/4814647
